# Complete Genome Sequences of Two Megasphaera elsdenii Strains, NCIMB 702410 and ATCC 25940

**DOI:** 10.1128/MRA.01430-18

**Published:** 2019-01-17

**Authors:** E. Anne Hatmaker, Dawn M. Klingeman, Kaela B. O’Dell, Lauren A. Riley, Beth Papanek, Adam M. Guss

**Affiliations:** a Biosciences Division, Oak Ridge National Laboratory, Oak Ridge, Tennessee, USA; b Bredesen Center for Interdisciplinary Research and Graduate Education, University of Tennessee—Knoxville, Knoxville, Tennessee, USA; University of Maryland School of Medicine

## Abstract

Here, we report the complete genome sequences of two Megasphaera elsdenii strains, ATCC 25940 and NCIMB 702410. M. elsdenii is an anaerobic bacterium capable of producing butanoate and hexanoate and is a member of the Negativicutes.

## ANNOUNCEMENT

Megasphaera elsdenii, an anaerobic Gram-negative bacterium found in the rumen of cattle and sheep, has the capability to produce butanoate as well as hexanoate, providing a foundation for longer-chain fatty acid biosynthesis (1). Due to the lack of genetic engineering tools for M. elsdenii, the underlying mechanism of hexanoate production is currently poorly characterized. Sequenced genomes provide valuable information regarding the underlying genetics of a strain. Therefore, M. elsdenii strains NCIMB 702410 and ATCC 25940 were sequenced to produce complete genomes to enable such comparisons. ATCC 25490 is also known as DSM 20460, for which a draft genome sequence is available under the GenBank accession number NC_015873 (2).

M. elsdenii strains NCIMB 702410 and ATCC 25940 were grown anaerobically in differential reinforced clostridial broth (HiMedia) at 37°C. High-molecular-weight genomic DNA was isolated from M. elsdenii ATCC 25940 and NCIMB 702410 cultures using a Genomic-tip 100/G kit (Qiagen, Valencia, CA), following the manufacturer’s protocol.

A Nextera XT library (Illumina, San Diego, CA) was prepared from M. elsdenii NCIMB 702410 genomic DNA, as described in the manufacturer’s protocol. The final library was validated on a Bioanalyzer instrument (Agilent) using a DNA7500 chip, and concentration was determined using a Qubit double-stranded DNA (dsDNA) broad-range (BR) assay kit (Invitrogen). Paired-end sequencing (2 × 301 bp) was completed using the Illumina MiSeq platform. Additional sequencing was performed by the Department of Energy’s Joint Genome Institute on both M. elsdenii NCIMB 702410 and ATCC 25940, using single-molecule real-time (SMRT) sequencing (3) on the PacBio RS II platform (Pacific Biosciences, Menlo Park, CA) to produce longer reads. Prior to SMRT sequencing, NCIMB 702410 genomic DNA underwent BluePippin size selection (Sage Science, Beverly, MA) to eliminate shorter fragments and to enrich sequencing of DNA fragments of >10 kb.

For the Illumina data quality control, reads were trimmed using Trimmomatic to remove the vectors and filtered within Geneious software (Auckland, New Zealand) based on quality, with an error probability rate of 0.05. For the PacBio data, RS II-filtered subreads were used. The genome of M. elsdenii NCIMB 702410 was assembled using the hybrid assembly capacity of SPAdes (4), with 2,118,666 forward and reverse short paired-end Illumina reads input as the main library and 114,534 PacBio RS II reads as an additional library. The assembly was trimmed and circularized, resulting in a single contiguous, circular chromosome of 2,566,193 bp, with coverage greater than 135×. The genome of M. elsdenii ATCC 25940 was assembled from 474,847 PacBio reads using Canu (5), an update of the Celera Assembler, and the resulting genome had over 200× coverage. Both M. elsdenii genomes have a single circular chromosome and were annotated using Rapid Annotations using Subsystems Technology (RAST) 2.0 (6). The genome of M. elsdenii ATCC 25940 is 2,478,842 bp in length, with a 52.8% GC content; therefore, the *N*_50_ value is 478,842, and the *L*_50_ value is 1. M. elsdenii NCIMB 702410 has a 2,566,193-bp genome and a 52.7% GC content; therefore, the *N*_50_ value is 2,566,193, and the *L*_50_ value is 1. Both genomes are predicted to encode 65 tRNAs and 21 rRNAs. The NCIMB 702410 genome was predicted to encode 2,280 coding regions, whereas the ATCC 25940 genome was predicted to encode 2,194 coding sequences.

### Data availability.

The complete genome sequence of Megasphaera elsdenii ATCC 25940 has been deposited in GenBank under the accession number CP027570, and the reads are available through the NCBI SRA under the accession number SRP156241. The complete genome sequence of M. elsdenii NCIMB 702410 has been deposited in GenBank under the accession number CP027569. The reads for strain NCIMB 702410 are available from the NCBI SRA under the accession number SRP152212.
